# Comparison of the prevalence and severity of nausea and vomiting in the first trimester between singleton pregnancies conceived from stimulated in vitro fertilization and frozen embryo transfer cycles

**DOI:** 10.1186/s12884-022-05072-5

**Published:** 2022-10-04

**Authors:** Evelyn Wong, Jennifer KY Ko, Raymond HW Li, Ernest HY Ng

**Affiliations:** Department of Obstetrics and Gynaecology, Queen Mary Hospital, The University of Hong Kong, 6/F Professorial Block, 102 Pokfulam Road, Pok Fu Lam, Hong Kong

**Keywords:** Nausea and vomiting in pregnancy, IVF, FET cycles, Stimulated cycles

## Abstract

**Objective:**

The objective of this prospective study is to compare the prevalence and severity of nausea and vomiting in the first trimester between singleton pregnancies conceived from stimulated in vitro fertilization (IVF) and frozen embryo transfer cycles (FET).

**Methods:**

All women were recruited at 6 weeks gestation and filled in the modified Pregnancy-Unique Quantification of Emesis and Nausea (PUQE) to document whether they had any experience of nausea and vomiting weekly till 12 weeks gestation. The primary outcome was the prevalence of nausea and vomiting and the secondary outcomes included severity of nausea and vomiting and pregnancy outcomes.

**Results:**

A total of 360 pregnant women were recruited and 171 were in the stimulated IVF group and 189 in the FET group. The overall return rate was 82.2% (81.8% in the stimulated IVF group and 82.5% in the FET group). Nausea and vomiting were worse in the FET group compared with the IVF group. There were significantly more women who felt nauseated or sick in the FET group (p value = 0.032 for week 11 and p value = 0.046 for week 12); significantly more women with a longer duration of nausea in the FET group (p value = 0.044 for week 7 and p value = 0.030 for week 8); significantly more women with more vomiting in a day in the FET group (p value = 0.042) and significantly more women with retching or dry heaves in the FET group (p value = 0.030 for week 8 and p value = 0.028 for week 11).

**Conclusion:**

Nausea and vomiting were significantly more prevalent and severe in the FET group when compared with the stimulated IVF group.

**Supplementary Information:**

The online version contains supplementary material available at 10.1186/s12884-022-05072-5.

## Introduction

Nausea and vomiting affect 70–80% of pregnant women and can range from mild “morning sickness” to severe hyperemesis gravidarum. The latter may cause fluid and electrolyte disturbance, weight loss, dehydration and deranged renal function requiring hospitalization [1].

The pathogenesis of nausea and vomiting is likely multifactorial but the exact mechanism remains unknown. Women on oral contraceptive pills or hormonal replacement therapy may experience nausea and vomiting, suggesting a possible role of oestrogen and progesterone [1]. Both oestrogen and progesterone are thought to contribute to nausea and vomiting by delaying gastric emptying [2]. Oestrogen stimulates the production of nitric oxide via nitrogen oxidase synthetase, which causes slowing of the gastric intestinal transit time and gastric emptying via smooth muscle relaxation [1]. One study has found that women with hyperemesis gravidarum have 26% higher serum levels of oestradiol compared to control [3]. In addition, it has been shown that nausea and vomiting are more severe in obese women who have a higher oestrogen level [3]. However, the effect of oestrogen in causing nausea and vomiting remained inconsistent as a review showed that only 5 out of 17 studies showed a positive relationship between nausea and vomiting and serum oestrogen level [4]. Progesterone causes nausea and vomiting by reducing smooth muscle contractility and affecting gastric emptying [1]. One study showed an additive effect of oestradiol and progesterone co-administration in delaying gastric emptying as demonstrated by increased gastric slow-wave rhythm [2].

During assisted reproduction, pregnancies can be achieved during stimulated in vitro fertilization (IVF) cycles or frozen embryo transfer (FET) cycles. In stimulated IVF cycles, women have embryo transfer following ovarian stimulation with gonadotrophins and serum oestradiol levels during early pregnancy are often much higher than those who conceive in FET cycles. In FET cycles, either in natural, letrozole-induced or hormone replacement cycles, serum oestradiol levels are closer to physiological range.

If serum oestradiol level is related to nausea and vomiting during early pregnancy, it is postulated that women who conceive in stimulated IVF cycles will experience more severe symptoms than those who conceive in FET cycles. However, there is still no study comparing the prevalence and severity of nausea and vomiting during early pregnancy between pregnancies conceived from stimulated IVF cycles and FET cycles. The objective of this prospective study aimed to compare the prevalence and severity of nausea and vomiting in the first trimester between pregnancies conceived from stimulated IVF cycles and FET cycles.

## Methods

### Study subjects

The prospective study was conducted between October 2016 to December 2018 in the Assisted Reproduction Unit at the Department of Obstetrics and Gynaecology, University of Hong Kong, Queen Mary Hospital, Hong Kong. It was approved by the Institutional Review Board of the University of Hong Kong/Hospital Authority Hong Kong West Cluster and was registered under the University of Hong Kong Clinical Trials Registry (registration number: HKUCTR-2124).

Infertile women undergoing stimulated IVF and FET were screened and recruited following their first ultrasound scan at 6 weeks of gestation. We recruited women under the age of 42 years, with a viable singleton intrauterine pregnancy in IVF cycles or FET cycles and those who had multiple pregnancy, underlying thyroid or gastric problems, molar pregnancy or those currently on traditional Chinese medicine were excluded. Written consent forms were signed after counselling.

All patients underwent their IVF treatment with ovarian stimulation using either the long gonadotrophin releasing hormone (GnRH) agonist protocol or fixed GnRH antagonist protocol as previously described [5]. During day 2–3 of the menstrual cycle, transvaginal ultrasound examination and serum oestradiol measurement were performed. Human menopausal gonadotrophin (hMG) (Menogon, Ferring GmbH, Kiel, Germany) or recombinant FSH (Puregon, Organon, Dublin, Ireland or Gonal F, Merck Serono S.p.A, Modugno, Italy) were started at a dose between 150 and 300 IU per day based on the antral follicle count and previous ovarian response, according to the standard operating procedures. Serial ultrasound scans with or without hormonal monitoring was performed to monitor ovarian response. Further dosage adjustments were based on the ovarian response. GnRH antagonist 0.25 mg/day (Orgalutran, Organon, Dublin, Ireland or Cetrotide®, Merck, Germany) was started on the sixth day of stimulation. When three leading follicles were ≥ 18 mm, 5000–10000IU human chorionic gonadotrophin (hCG) (Pregnyl®, Organon, Oss, the Netherlands) or 250 mg Ovidrel® (Merck Serono S.p.A, Modugno, Italy) was given to trigger final oocyte maturation. Oocyte retrievals under the guidance of transvaginal ultrasound were scheduled 34–36 h later. A maximum of two embryos or blastocysts were transferred 2 or 5 days respectively after oocyte retrieval. Single embryo transfer was advised in women less than 35 years old. Excess good quality embryos were frozen for subsequent transfer.

The details of the freezing and thawing protocols were reported previously [6]. In ovulatory women, natural cycles were used for FET. Luteinizing hormone (LH) surge was defined as an elevation of the LH to 2 times the level of the average of the previous 3 days and the absolute level of the LH should be greater than 20 IU/L. It was determined by serial blood tests, and FET was performed on the third day after LH surge for day 2 embryos and on the sixth day for blastocysts. For anovulatory subjects, FET was carried out in either letrozole-induced or hormone replacement cycles. A maximum of two frozen embryos or blastocysts were allowed to be transferred in any one FET cycle.

Pregnancy test was performed 18 days after hCG trigger in stimulated IVF cycles, or LH surge or starting vaginal progesterone in FET cycles. All women with a positive pregnancy test were recruited following their first ultrasound scan at 6 weeks of gestation. They filled in a questionnaire to document whether they had any nausea and vomiting weekly till 12 weeks of gestation. The modified Pregnancy-Unique Quantification of Emesis and Nausea (PUQE) index was used to quantify the symptoms of nausea and vomiting [7] (Appendix). It included the amount of time the respondents felt nauseated or sick in a day, the number of times they vomited and the number of times they had retching or dry heaves without vomiting during a day. Women were referred for antenatal care after 12 weeks of gestation.

### Outcome measures

The primary outcome of this study was the prevalence of nausea and vomiting. The secondary outcomes included severity of nausea and vomiting which was shown by the frequency and number of times of nausea and vomiting, and whether seeing a doctor or hospitalization was necessary.

Other secondary outcomes were the miscarriage rate, complications of pregnancy, sex and weight of the baby Complications of pregnancy included small for gestational age, which was defined as fetal weight < 10th centile for gestation, low birth weight which was defined as birth weight < 2.5 kg; preterm delivery which was defined as delivery of fetus before 37 completed weeks of gestation, gestational hypertension which was defined as raised diastolic blood pressure (DBP) > = 90mmHg consecutively 4 h apart or DBP > = 110mmHg on any one occasion), pre-eclampsia which was defined by gestational hypertension and gestational proteinuria (urine protein > = 0.3 g/day or urine protein / creatine ratio > = 30) with onset after 20 completed weeks of gestation, with both returned normal by 3 months postpartum, gestational diabetes which was defined by a 75 g oral glucose tolerance test (OGTT) during pregnancy showing fasting glucose > = 5.1mmol/L, 1 h glucose > = 10.0mmol/L or 2 h glucose > = 8.5mmol/L, which returned normal 8 weeks postpartum, and perinatal death which was defined as stillbirth or early neonatal death.

### Sample size calculation

It has been reported that the prevalence of nausea and vomiting in natural pregnancy was around 70% [1]. We hypothesized that the prevalence of nausea and vomiting in pregnant women from stimulated IVF cycles was 85%. To achieve statistical comparison with 80% power and a two-sided 5% level of statistical significance, 242 women (121 in each arm) were needed to show the anticipated difference of 15% (70% versus 85%) between stimulated IVF and FET cycles. To account for 25% drop out rate, at least 150 women in each arm were aimed for.

### Statistics

Demographic and clinical characteristics were summarized with counts (percentages) for categorical variables, mean (standard deviation [SD]) for normally distributed continuous variables, or median (25th – 75th percentile) for non-normally distributed continuous variables. Demographic features and outcomes of the two study groups were compared. Chi-square test or Fisher’s exact test were used for categorical variables. Mann-Whitney U test was used to compare the continuous variables between the two groups where appropriate. Statistical analyses were carried out using IBM SPSS Statistics, version 25 (IBM Corporation, New York, USA). A two-sided *P* < 0.05 was taken as statistically significant.

## Results

A total of 360 pregnant women were recruited: 171 in the stimulated IVF group and 189 in the FET group. The mean number of questionnaires returned for each week was 296 (140 in the stimulated IVF group and 156 for the FET group). The overall return rate was 82.2% (81.8% in the stimulated IVF group and 82.5% in the FET group).

Table 1 showed the baseline characteristics in the stimulated IVF group and FET group.

**Table 1:** Baseline characteristics and secondary outcome of women undergoing stimulated in vitro fertilization (IVF) and frozen embryo transfer cycles (FET)

Baseline Characteristics			
	FET group (n = 189)	Stimulated IVF group (n = 171)	P value
**Age of women (years)**	36 (33–37)	36 (34–38)	0.387
**Body mass index (kg/m2)**	21.94 (20.37–23.97)	22.72 (20.55–24.46)	0.08
**Infertility cause**			
Tubal	30	29	0.781
Male	67	62	0.873
Unexplained	37	35	0.833
Anovulation	8	8	0.838
Mixed	40	23	0.0544
Others	7	14	0.070
**Infertility duration (years)**	4 (3–6)	4 (3–6)	0.360
**Serum oestradiol on trigger day in stimulated IVF or LH surge in FET**	960 (796–1197)	6425 (3543–10,212)	< 0.0001
**Stage of embryos replaced**			0.099
Cleavage stage	152	116	
Blastocyst	37	55	
**Nulliparous**	140 (74.1)	167 (97.7%)	< 0.0001
**Nausea and vomiting in last pregnancy**			0.567
Yes	0 (0%)	0 (0%)	
No	56 (29.6%)	58 (33.9%)	
Not applicable	133 (70.4%)	113 (66.1%)	
**Secondary outcome**			
** Pregnancy Outcomes**			0.506
Miscarriage	23 (12.2%)	21(12.3%)	
Ectopic pregnancy	0 (0%)	2 (1.17%)	
Livebirth	166 (87.8%)	148 (86.6%)	
** Mode of delivery**			< 0.001
Normal spontaneous delivery	49 (29.5%)	45 (30.4%)	
Assisted delivery	18 (10.8%)	20 (13.5%)	
Lower segment Caesarean section	99 (59.6%)	82 (55.4%)	
Assisted breech delivery	0 (0%)	1 (0.7%)	
** Sex of baby**			< 0.001
Female	75 (45.2%)	83 (56.1%)	
Male	91 (54.8%)	65 (43.9%)	
** Complications of pregnancy**			
Small for gestational age	4 (2.4%)	3 (2.0%)	0.786
Low birth weight	21(12.7%)	24 (16.2%)	0.439
Preterm delivery	3(1.8%)	6 (4.1%)	0.255
Pre-eclampsia and gestational hypertension	5 (3.0%)	4 (2.7%)	0.832
Gestational diabetes	31 (18.7%)	23 (15.5%)	0.394
Perinatal mortality	2 (1.2%)	5 (3.4%)	0.209
Placenta praevia	2 (1.2%)	5 (3.4%)	0.209
Cervical incompetence	0 (0%)	0 (0%)	0
** Birth weight (gram)**	3060 (2770–3320)	3018 (2635–3245)	0.727

No significant differences were found in age of women, body mass index, causes/duration of infertility, stage of embryos replaced and history of nausea and vomiting in the previous pregnancy between the two groups. There were significantly more nulliparous women in the stimulated IVF group. Serum oestradiol level was higher in the stimulated group and this was due to ovarian stimulation. In the FET group, 144 women had natural cycles, 42 had artificial cycles and 3 had clomid induced cycles. Table 1 also showed other secondary outcomes between the stimulated IVF and FET groups. There was no significant difference in occurrence of pregnancy outcomes, complications of pregnancy and birth weight between the two groups. There were significantly more female babies in the stimulated IVF group than the FET group.

**Fig. 1:**
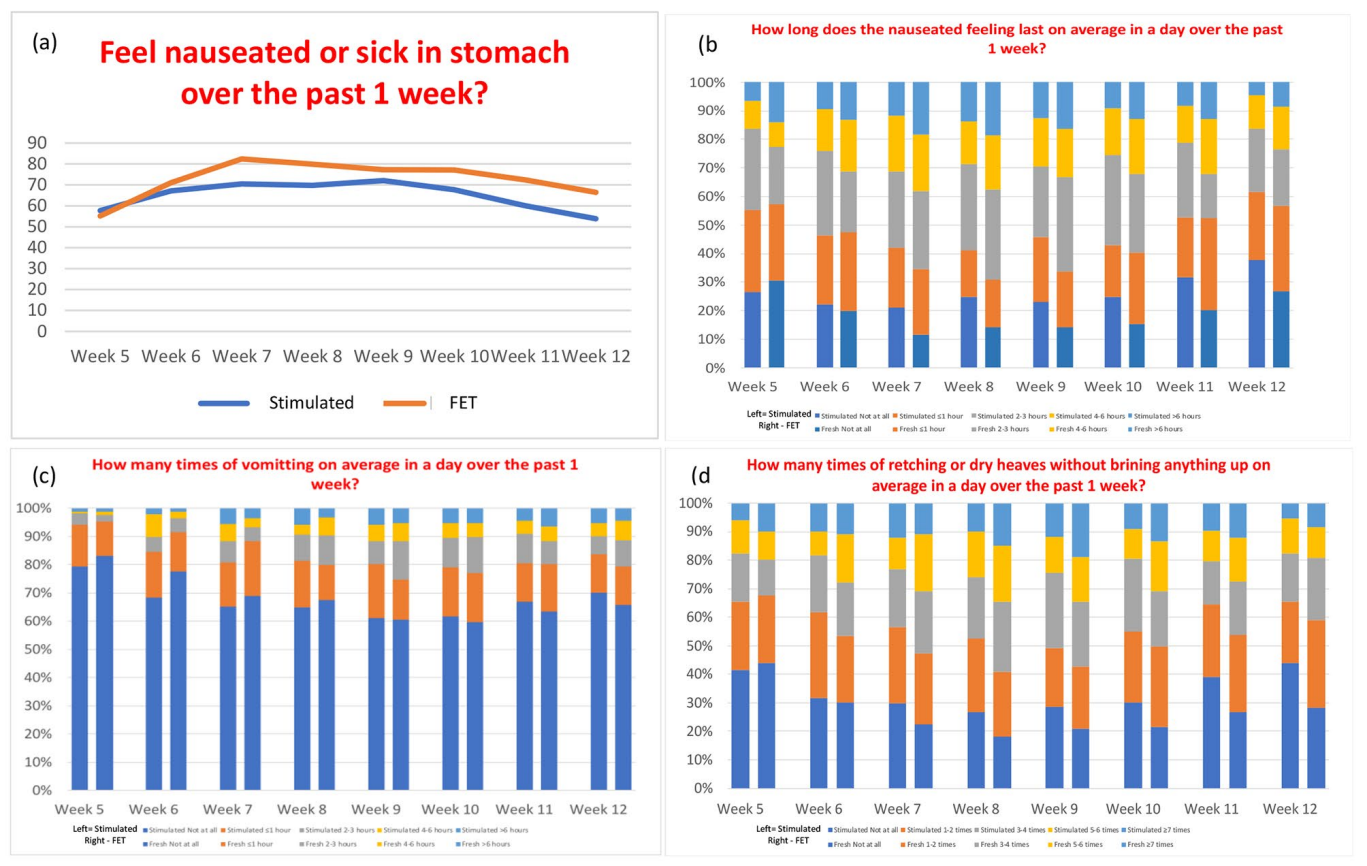
Severity of nausea and vomiting in stimulated IVF and FET cycles. (a) Feeling nauseated or sick from week 5 to week 12 of pregnancy. (b) Duration of nauseated feeling in a day from week 5 to week 12 of pregnancy. (c) Number of times of vomiting on average in a day from week 5 to week 12 of pregnancy. (d) Number of times of retching or dry heaves on average in a day from week 5 to week 12 of pregnancy. (b,c,d) Left bar of each week represents stimulated IVF cycle and right bar represent FET cycle

Figure 1a showed the number of women who felt nauseated or sick over different weeks of gestation in the stimulated IVF and FET groups. During week 11 and 12 of gestation, there were significantly more women who felt nauseated or sick in the FET group (p value = 0.032 for week 11 and p value = 0.046 for week 12). During week 7 and 8, there were significantly more women with a longer duration of nausea in the FET group (p value = 0.044 for week 7 and p value = 0.030 for week 8) (Fig. 1b). During week 6, there were significantly more women with more vomiting in a day in the FET group (p value = 0.042) (Fig. 1c). During week 8 and 11, there were significantly more women with retching or dry heaves in the FET group (p value = 0.030 for week 8 and p value = 0.028 for week 11) (Fig. 1d).

We performed propensity matching score with a matching threshold of 0.085 (corresponding to 0.5 standard deviations), to match for woman’s age, BMI, fetal gender and parity. There was no significant difference between the matched pairs (p = 0.248) by McNemar test.

We have also evaluated the effect of different factors affecting the severity of nausea and vomiting. Multivariate logistic regression analysis showed that younger women were more likely to experience nausea and vomiting at any time during the study period (B -0.263, Exp(B) 0.769, 95% CI 0.681–0.868) Parity, history of nausea and vomiting during previous pregnancy and the sex of the baby did not significantly affect the severity of nausea and vomiting.

## Discussion

To the best of our knowledge, this is the first prospective study comparing the prevalence and severity of nausea and vomiting in between pregnancies from stimulated IVF cycles and FET cycles. Our study showed that nausea and vomiting was more prevalent in the FET group compared with the stimulated IVF group which was in contrary to our hypothesis.

Oestrogen and progesterone had been thought to be associated with increase in nausea and vomiting in early pregnancy due to reduction in gastric emptying and intestinal transit time. However, recent studies showed that women with hyperemesis experience faster motility rates [8]. Moreover, there were no published studies that found a relationship between the severity of hyperemesis and serum oestrogen level. Previous studies showed a lack of relationship between progesterone and severity of hyperemesis [9–11]. In addition, for pregnancies where progesterone was given for luteal phase support, an increased severity in hyperemesis or nausea and vomiting was not observed [8].

This finding of our study could be explained by the fact that there are many factors that can affect the severity of nausea and vomiting in pregnancy. Our study showed that younger women were likely to experience nausea and vomiting. This concurred with findings from other previous studies [7, 12, 13]. Multiparity had been shown to be a risk factor for more severe nausea and vomiting during pregnancy [14, 15] and indeed there were significantly more multiparous women in the FET group. This might be partly due to the exclusion of parous women from public funding for further stimulated IVF cycles in our locality.

Another possible reason to explain more nausea and vomiting in FET group could be related to beta-human chorionic gonadotropin (β-hCG) level. One study showed that there is significantly higher serum β-hCG level and increment in the FET group compared with the stimulated group [16]. β-hCG is associated with more severe vomiting since women with hydatidiform moles and twin pregnancy experience more vomiting [10, 17]. 

Propensity matching analysis was performed to minimise the heterogeneity of patients by matching for woman’s age, BMI, fetal gender and parity and showed no statistical significance between the stimulated IVF group and the FET group. This may be explained by a reduction of sample size after matching analysis since only 118 pairs of subjects could be matched. This was because there were twin pregnancies with discordant gender hence no propensity was calculated, there were patients with miscarriage hence fetal gender was unknown and some had no available close matching.

The strength of this study was the prospective design. The use of the modified PUQE index, a validated symptom quantification tool, allowed an objective measure of the severity of nausea and vomiting which allowed a better interpretation of differences between both groups. The questionnaire completion and return rates were up to 82%. We recruited women when they are 6 weeks pregnant, hence we will not miss any early pregnancy vomiting.

In this study, we did not collect data on the use of any drugs during the study period. Potential use of anti-emetics or over-the-counter drugs could have affected the severity of nausea and vomiting hence affecting the significance of the results. Majority of our patients had mild symptoms of nausea and vomiting and only very few required hospitalization. This shows that the severity of nausea and vomiting may not be clinically significant since the majority of them only experienced mild symptoms that may not have affected their quality of life. However, we did not ask our patients to fill in questionnaire assessing the effect nausea and vomiting on their quality of life. As our patients were asked to fill in the questionnaire every week and return them every 2 weeks, we were unable to know when exactly they filled it in and potentially there could be recall bias. In addition, we did not measure blood for serum oestradiol, progesterone and hCG levels at various gestation between the two groups.

## Conclusion

In this study, nausea and vomiting in the first trimester were significantly more prevalent and severe in singleton pregnancies conceived from FET cycles when compared with those from stimulated IVF cycles.

## Electronic supplementary material

Below is the link to the electronic supplementary material.

Supplementary Material 1

## Data Availability

The datasets used and/or analysed during the current study are available from the corresponding author on reasonable request.

## Abbreviations

IVF: in vitro fertilization.
FET: frozen embryo transfer cycles.
PUQE: Pregnancy-Unique Quantification of Emesis and Nausea.
GnRH: gonadotrophin releasing hormone.
hMG: Human menopausal gonadotrophin.
hCG: Human chorionic gonadotrophin.
LH: Luteinizing hormone.
DBP: diastolic blood pressure.
OGTT: oral glucose tolerance test.
CI: confidence interval.
