# Supplementary material for: Comparison of the prevalence and severity of nausea and vomiting in the first trimester between singleton pregnancies conceived from stimulated in vitro fertilization and frozen embryo transfer cycles
Source: BMC Pregnancy Childbirth. 2022 Oct 4;22:746. doi: 10.1186/s12884-022-05072-5 (PMC9531374; doi:10.1186/s12884-022-05072-5)
Supplement: Supplementary file 1 — Supplementary Material 1 [file 12884_2022_5072_MOESM1_ESM.docx]

**Appendix**

**Questionnaire for Nausea and Vomiting during early pregnancy**

Date: From _______________________(dd/mm/yy) to _______________________(dd/mm/yy)

1. On average in a day over the past 1 week, for how long do you feel nauseated or sick to your stomach?

Not at all ≤1 hour 2-3 hours 4-6 hours >6 hours

(1) (2) (3) (4) (5)

1. How long does the nauseated feeling last on average in a day over the past 1 week?

Not at all ≤1 hour 2-3 hours 4-6 hours >6 hours

(1) (2) (3) (4) (5)

1. On average in a day over the past 1 week, how many times do you vomit or thrown up?

Did not throw up 1-2 times 3-4 times 5-6 times ≥7 times

(1) (2) (3) (4) (5)

1. On average in a day over the past 1 week, how many times do you have retching or dry heaves without brining anything up?

None 1-2 times 3-4 times 5-6 times ≥7 times

(1) (2) (3) (4) (5)

1. Did you require taking any herbs or traditional Chinese medicine for your nausea or vomiting over the past 1 week?

(1)Yes (2) No

1. Did you need to see a doctor for your nausea and vomiting over the past 1 week?

(1) Yes (2) No

1. Were you hospitalized for your nausea and vomiting over the past 1 week?

(1) Yes (2) No

End of Questionnaire
